# Single Nucleotide Polymorphisms: A Window into the Informatics of the Living Genome

**DOI:** 10.4236/abb.2014.57073

**Published:** 2014-06

**Authors:** Georgia M. Dunston, Tshela E. Mason, William Hercules, James Lindesay

**Affiliations:** 1 Department of Microbiology, Howard University, Washington DC, USA; 2 National Human Genome Center, Howard University, Washington DC, USA; 3 Computational Physics Laboratory, Howard University, Washington DC, USA

**Keywords:** SNPs, Genomic Information, Genodynamics, Biophysical Metrics, Allelic Energies

## Abstract

Nested in the environment of the nucleus of the cell, the 23 sets of chromosomes that comprise the human genome function as one integrated whole system, orchestrating the expression of thousands of genes underlying the biological characteristics of the cell, individual and the species. The extraction of meaningful information from this complex data set depends crucially upon the lens through which the data are examined. We present a biophysical perspective on genomic information encoded in single nucleotide polymorphisms (SNPs), and introduce metrics for modeling information encoded in the genome. Information, like energy, is considered to be a conserved physical property of the universe. The information structured in SNPs describes the adaptation of a human population to a given environment. The maintained order measured by the information content is associated with entropies, energies, and other state variables for a dynamic system in homeostasis. “Genodynamics” characterizes the state variables for genomic populations that are stable under stochastic environmental stresses. The determination of allelic energies allows the parameterization of specific environmental influences upon individual alleles across populations. The environment drives population-based genome variation. From this vantage point, the genome is modeled as a complex, dynamic information system defined by patterns of SNP alleles and SNP haplotypes.

## 1. Introduction

The completion of the Human Genome Project (HGP) in 2003 introduced a new 21^st^ century knowledge-base for decoding biology, and the science of genomic information structured in DNA sequence variation. In this short report on single nucleotide polymorphisms (SNPs), we explore a biophysical perspective on the genome as the most sophisticated living “information and communication system” known to mankind. As such, it is expressed in and through all levels of existence, from the invisible world of quantum physics through the visible biological hierarchy of molecules, cells, tissues, organs, and systems integrated in whole individuals, to the limitless, global diversity of all human populations that constitute humanity. The early recognition of SNPs as common variants widely distributed in the genome provoked broad interests in their utility as genomic markers. The rapid development of high throughput DNA sequencing accompanied by bioinformatics tools and technologies for analyzing and managing these massive datasets have revolutionized human genome studies, thrusting 21^st^ century DNA sequence-based biology into the data-driven arena of “big science”. The abundance of easily ascertained SNPs, which occur approximately every 600 – 1000 bp across the genome, made them the DNA marker of choice in genetic epidemiology, genome-wide association studies (GWAS), DNA sequence variation research, pharmacogenomics, anthropological and population genetics. Although other types of genome variation (e.g. insertions/deletions, microsatellites, copy number variants, and epigenetic markers) have been employed in disease studies, SNPs have been the most useful and widely applied markers in biomedical research and behavioral science. The advent of genome-wide population-based variation resources, such as the Haplotype Map, has changed the way we conceptualize human variation, categorize human populations, and conduct population studies. The distribution of genome variation in large continental population groups has posed major challenges to the authenticity and biological integrity of institutionalized social constructs of human races. Online public availability of SNP alleles and SNP haplotype data for global populations, defined geographically by continent of origin, have forged a new era in population genetics, offering unprecedented opportunities to investigate adaptive forces that have shaped patterns of common variation in natural populations.

## 2. The Human Genome Model of Life

Recently, the Howard University National Human Genome Center (NHGC) Biophysics Research and Development Group applied first principles of thermodynamics and statistical physics in studying the informatics of SNPs as dynamic sites in the genome [1]. We introduced a biophysical metric for interrogating the information encoded in the genome. We assert that SNPs are dynamic sites in the genome of a population and that information structured in these common variants describes the adaptation of a human population to a given environment. The maintained order parameterized by the information content (IC) is related to state variables for a dynamic system in homeostasis. Entropy *S* is a measure of the disorder (noisiness) of a distribution of occurrences, expressed as:

(1) $$S=-\sum p_{j}\log p_{j}$$

where *p_j_* is the probability of the occurrence of type *j*.

In contrast, information *I* is a measure of maintained order, which can be parameterized by the difference between the entropy of a system of maximum disorder and that of the entropy of the given system,

(2) $$I=S_{\max}-S.$$

The information is contained both in individual SNPs, as well as, those combinations of SNPs in linkage disequilibrium (*i.e*. SNP haplotypes), which are maintained throughout generations.

Our normalization of this information metric (*NIC*) allows for comparison of SNP haploblocks across different sites in the genome and across different populations. *NIC* is defined as,

(3) $$\mathrm{NIC}=I/S_{\max}.$$

**Figure 1:**
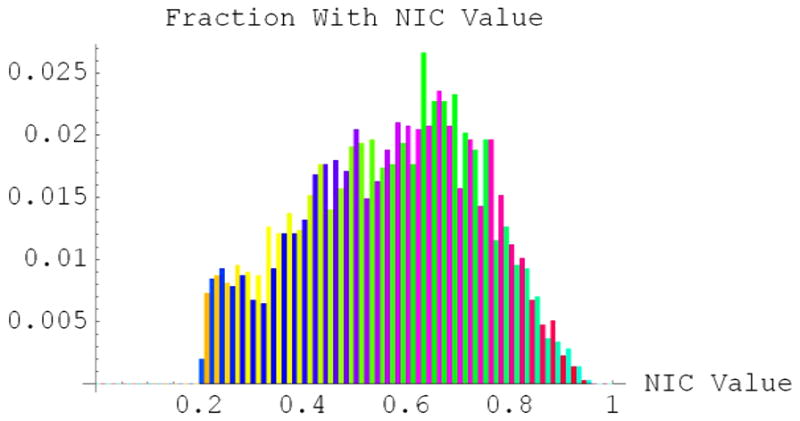
The distribution of *NIC* values for the HLA-DR region on chromosome 6 in an African-American population from the Southwest, US. The height of each bar is the fraction of all *NIC* values that fall within the channel given by the width of the bar. Colors are chosen by an algorithm to provide contrast in viewing adjacent channels.

The 21^st^ century emergence of genomic medicine is shifting the paradigm in medical research from population phenotypes to individual genotypes. In population genetics and epidemiology, populations are often defined by the most common traits or most frequent allele(s) in the group. This definition poses difficulties in categorizing individuals in the population who do not fit the phenotype. Our work demonstrates the promise of defining populations by their SNP haplotype frequency distributions, a quantitative genomic population metric that incorporates the allelic contribution of all persons in the population rather than discrete alleles and/or most common traits in the population. NIC values range from 0 to 1. In Figure 1, a distribution of *NIC* values in the human leukocyte antigen (HLA)—Disease Related (DR) region of the major histocompatibility complex shown below for African-Americans in Southwest, USA, we found that SNP haploblocks with low *NIC* values contained regulatory elements involved in innate immunity, which allows for rapid adaptation to environments, whereas SNP haploblocks with high *NIC* were in genic regions [1].

## 3. Dynamics of Genome-Environment Homeostasis

Our biophysical perspective on genomic information facilitates the characterization of the dynamic impact of the environment on genome variation. We have translated SNP haplotype data into entropic measures of *NIC* that correlate with genomic energy units (GEUs) [2]. The latter biophysical metric can be used to assess the interplay of maintained statistical genomic variation with the environmental bath within which stable populations exist. This allows the development of the analogous “thermodynamics”, which we term “genodynamics”. Genodynamics characterizes the state variables for whole genomes that are stable under stochastic environmental stresses. Our model facilitates the determination of both population averaged state variables as well as genomic energies of individual alleles and their combinations (*i.e*. haplotypes). The determination of individual allelic energies then allows the parameterization of specific environmental influences upon shared alleles across populations in varying environments. A stable genomic free energy trades off low genomic energy *U* (parameterizing genomic conservation and increased order) against high genomic entropy *S* (parameterizing genomic variation), where *T_E_* is an environmental potential which drives variation. The genomic free energy is given by the equation,

(4) $$F_{\mathrm{Genome}}=U-T_{E}S.$$

Individual allelic and haplotype energies are directly related to *p_j_* which is the probability of occurrence of allele or haplotype *j*. The metrics defining the dynamics should prove particularly relevant to characterizing individual genotypes in the emergent era of personalized/precision genomic medicine. Human migration has provided us with natural laboratories that map whole genome adaptation to environmental stresses (like malaria, UV light, lactose, etc.), allowing the examination of individual genomes within their environment. Our use of GEUs as a biophysical metric in DNA sequence analysis has provided new insights into the foundations of population differences expressed in and through SNP allele and haplotype variation. Viewing genomic big data through the lens of theoretical physics has allowed us to develop energy measures for modeling genome-environment interactions.

Endowed with the capacity for self recognition and unlimited self replication, the human genome, encodes and expresses principles of life and living systems, not unlike those displayed in stem cells and cancer cells. The non-random, wide distribution of SNPs provokes intriguing questions on the function of this system of natural variation. With the preponderance of SNPs in non-protein coding regions of the human genome, a series of papers from the Encyclopedia of DNA Elements (ENCODE) Project Consortium published in *Nature* in 2012, showed that individuals harbor considerably more regulatory sequences in non-coding compared to protein-coding variants. These data support the likelihood of a common regulatory mechanism for SNPs. From our perspective, SNPs are dynamic sites correlated with patterns of conserved versus variable sequences, with the environment as the driving force for variation. Our model allows us to directly study genome-environment interactions and the effect of the environment on a given genotype for an individual as well as the population. Genodynamic studies of SNP alleles and haplotypes in non-protein coding regions should be instructive for modeling and testing the impact of the environment on regulatory mechanisms.

## 4. Human Genome Variation and Human Identity

Despite phenomenal gains from the HGP in deciphering the pathophysiology of common diseases and significant improvements in biological knowledge-based therapies, disadvantaged and minority communities still carry a disproportionate burden from common complex diseases, characterized as health disparities.

SNPs are powerful markers for probing and assessing aspects of human identity that are common to and shared by all individuals and peoples. We now have a mechanism to investigate the dynamics of how a whole genome (and its parts) adapts to its environment. We submit that parameterization of genomic energy associated with information structured in sequence variation can be insightful in testing models of health and disease. An analysis of SNPs as complex dynamical systems can provide tremendous insight on the extent to which genomes differ from cell to cell, person to person, family to family, and from population to population. Inquiring minds want to know: 1) What is the link between genes and behavior? 2) How does our genotype interact with our environment to produce behavior? 3) What are the societal and ethical implications of SNP discoveries in the non-protein coding regions of the genome? Towards this end, SNP databases may be queried for patterning in regions of comparable genomic energies for correlations with well-defined instruments for phenotyping psychological parameters, as well as variation in host response to well-defined psychological stressors.

## 5. Conclusion

One of the first lessons learned from sequencing the human genome was that we are all uniquely the same. This means that all of humanity is the expression of one incredibly diverse genome. We recognize SNPs as common variants strategically enmeshed throughout the genome as dynamic sites involved in regulation and expression of whole genome homeostasis.
